# ‘What’s in a name? Fit-for-purpose bacterial nomenclature’: meeting report

**DOI:** 10.1099/ijsem.0.006844

**Published:** 2025-07-22

**Authors:** Sheila Patrick, Laura Filkins, Markus Goker, Nicola Holden, Paul A. Hoskisson, Angelika Kiepas, Conor Meehan, Mark Pallen, Leighton Pritchard, Amanda L Suchanek, Iain Sutcliffe, Martha E Trujillo, Nicholas Tucker, Jake D. Turnbull, Susan Butler-Wu

**Affiliations:** 1 Wellcome-Wolfson Institute for Experimental Medicine, School of Medicine, Dentistry and Biomedical Sciences, Queen’s University Belfast, 97 Lisburn Rd, Belfast BT9 7BL, UK; 2 Department of Pathology, University of Texas Southwestern Medical Center, Dallas, USA; 3 Leibniz Institute DSMZ-German Collection of Microorganisms and Cell Cultures GmbH, Inhoffenstraße 7B 38124 Braunschweig Science Campus Braunschweig-Süd, Braunschweig, Germany; 4 Scotland’s Rural College Craibstone, Aberdeen, AB21 9YA, UK; 5 Strathclyde Institute of Pharmacy and Biomedical Sciences, University of Strathclyde, 161 Cathedral Street, Glasgow, G4 0RE, UK; 6 School of Science and Technology, Nottingham Trent University, Clifton Campus, Nottingham, NG11 8NS, UK; 7 Quadram Institute, Norwich Research Park, Norwich, NR4 7UA, UK; 8 bioMérieux, 1201 South 4800 West, Salt Lake City, Utah, 84104, USA; 9 Applied Sciences Department, Northumbria University, Newcastle upon Tyne, UK; 10 Departamento de Microbiología y Genética, Campus Miguel de Unamuno, University of Salamanca, 37007 Salamanca, Spain; 11 Allied Health Sciences, University of Suffolk, University Avenue, Ipswich, Suffolk, IP3 0FS, UK; 12 The National Collection of Type Cultures, UKHSA Culture Collections, 61 Colindale Avenue, Colindale, London, NW9 5EQ, UK; 13 Clinical Pathology and Clinical Microbiology, Keck School of Medicine, University of Southern California, LA General Medical Center, Los Angeles, USA

**Keywords:** bacterial nomenclature, infectious disease, infection diagnostics, food safety, phylogenomics, plant pathology

## Abstract

Rapid and economical DNA sequencing has resulted in a revolution in phylogenomics. The impact of changes in nomenclature can be perceived as an absolute necessity of scientific rigour, coupled with the slight inconvenience of needing to re-learn names. In relation to practical aspects of microbiology, for example, infectious disease diagnosis, there may, however, be potential dangers. Historically, prokaryote classification has been based on multiple metabolic, physiological, biochemical and descriptive characteristics combined with the environmental source. Whole-genome sequence data have transformed our ability to determine evolutionary relationships. In addition, metagenomic and metataxonomic sequencing have resulted in the discovery of novel microbes, many of which are yet to be cultured. As a result, occasional name changes and additional prokaryote discoveries have accelerated at an unprecedented pace. Herein is a report of a Microbiology Society supported meeting of representatives of the communities of specialist taxonomists, phylogeneticists and applied microbiologists. Discussion included: recent advances in phylogenomics and the potential impact of nomenclature change on practical microbiology, e.g. plant pathology, food security, industrial microbiology, clinical microbiology and infectious diseases; the need, or lack thereof, for wider consideration and consultation prior to nomenclature change proposals which impact on practical microbiology; the application of the intricate and highly necessary rules of prokaryote nomenclature, which sometimes appear unfathomable to the non-specialist; and genome-based phylogenomics and the relationship with the International Code of Nomenclature of Prokaryotes. The meeting resulted in the formation of the Ad Hoc Committee for Mitigating Changes in Prokaryotic Nomenclature under the auspices of the International Committee on Systematics for Prokaryotes.

## Introduction

Rapid and increasingly economical whole-genome sequencing has led to an unprecedented upheaval in the number of proposed prokaryote names. With more than two million prokaryotic genome sequences publicly available, many analyses indicate that current microbial taxonomy and nomenclature inherit elements of historical biological classification that do not correspond to quantitative clustering of prokaryotes on the basis of genome comparisons [1,3]. The impact of such rapid changes, including the revision of existing classifications, on practical aspects of Microbiology, however, can be perceived as ranging anywhere from inconvenient to potentially dangerous in relation to infectious diseases/clinical microbiology [4].

The Microbiology Society (https://microbiologysociety.org/) supported a focused meeting in September 2023 to explore the potential impact of nomenclature changes on the disciplines of practical microbiology, including plant pathology, food security, industrial microbiology and infectious diseases, and covered aspects of reclassification and the discovery of novel bacteria in a multi-disciplinary format.

Presentation topics at the meeting included the following key areas: (1) the current rules governing prokaryotic nomenclature, (2) alternative methods to cope with the volume of DNA sequence data and new discoveries of microbes and (3) examples of the impact of nomenclature change on practical microbiology. These were followed by focused small discussion groups to address specific questions, designed to prompt discussion and generate debate among attendees, as detailed in Box 1.

Box 1: **Questions provided to stimulate the discussion session**

1. Although [Insert new name] is validly published, anyone working with ‘this’ microbe is not obliged to accept the new name and may continue to use the previous one. Without regulation, the list of validly published names is meaningless to the non-taxonomist.
2. What is the point of nomenclature without consensus on which name should be used?
3. The process of name change proposals relies on a small number of individuals, authors, reviewers and editors. How could wider scrutiny by potential end-users of name change proposals be introduced?
4. For new name proposals, there is consideration of the correct use of ‘taxonomic’ Latin. What else should be considered? e.g. ease of pronunciation
5. Is the fungal system of recommended names translatable to bacteriology?
6. How can end-users have oversight of nomenclature changes in diagnostic (and other) databases?
7. Can a microbe be defined by the genome sequence? Does the genome equal the microbe?
8. Is a genome sequence essential for species description?
9. How do we deal with metagenome-assembled genomes taxonomically in the context of the list of approved names and ICSP?
10. Seq-code or *Candidatus* for yet-to-be cultured microbes – pros and cons

To our knowledge, this meeting was the first such meeting to facilitate and report on a mutual exchange of perspectives between the communities of specialist taxonomists, phylogeneticists and practical microbiologists. This gathering has spurred an ongoing inter-disciplinary dialogue including the formation of the Ad Hoc Committee for Mitigating Changes in Prokaryotic Nomenclature.

## Nomenclature changes and the code

Background on the governance of changes in prokaryote nomenclature as well as an overview of International Code of Nomenclature of Prokaryotes (ICNP) was provided by Iain Sutcliffe [at that time outgoing chair of the International Committee on Systematics for Prokaryotes (ICSP)], Markus Göker (Secretary of the ICSP’s Judicial Commission) and Martha Trujillo (Editor-in-Chief of the International Journal of Systematic and Evolutionary Biology). These presentations highlighted the correct definition and the meaning of the ‘valid publication’ of a prokaryotic name (Box 2). An example of a species with multiple validly published names is provided in Box 3 to illustrate that the correct name can be dependent on the opinion of the user and is not necessarily the most recently validly published name. Critically, these presentations addressed the evident widespread misconception that the most recently validated published name replaces previously validly published names and that its use is, therefore, mandated by the ICNP. In addition, Markus Göker presented a roadmap for taking forward the issues discussed during the meeting.

Box 2: **(Oren *et al.*, 2023)**

- Criteria for the valid publication of a name under the ICNP.
- There must be a correctly formed name and an etymology.
- There must be a description of the organism or a reference to a description elsewhere.
- A type strain must be assigned for names of new species or subspecies and new combinations (comb. nov.)
- Cultures of the type strain must be deposited, without restrictions, in two culture collections in two different countries.
- The new name must be published in the International Journal of Systematic and Evolutionary Microbiology (https://www.microbiologyresearch.org/content/journal/ijsem)

Box 3: **‘Correct names’ in prokaryotic nomenclature**

Principle 6 of the ICNP states that the ‘correct name of a taxon is based upon valid publication, legitimacy and priority of publication’, while Principle 8 states that ‘Each taxon with a given circumscription, position and rank can bear only one correct name, i.e. the earliest that is in accordance with the Rules of this *Code*’.

Where species have been reclassified into different genera, it is usual that these species have more than one name that is validly published and legitimate. Which name is considered the correct name, thus, depends on the taxonomic opinion of the user as to what is the appropriate position (classification in which genus), as shown in the following example:

Three homotypic synonyms that are names of the same species represented by the type strain C7^T^=ATCC 6939^T^=DSM 20307^T^=NCTC 1621^T^ (and other culture collection deposits).

*Corynebacterium equi* Magnusson 1923 [47]

- Validly published and legitimate; the correct name if the taxonomist’s opinion is that the appropriate position of the species is in the genus *Corynebacterium* Lehmann and Neumann 1896 [47].

*Rhodococcus equi* (Magnusson 1923) Goodfellow and Alderson 1977 [47]

- Validly published and legitimate; the correct name if the taxonomist’s opinion is that the appropriate position of the species is in the genus *Rhodococcus* Zopf 1891 [47].

*Prescottella equi* (Magnusson 1923) [48]

- Validly published and legitimate; the correct name if the taxonomist’s opinion is that the appropriate position of the species is in the genus *Prescottella* Sangal *et al*. 2022 [48].

When deciding which is the correct name to use, the user may choose to use an older validly published and legitimate name over the result of a more recent classification to maintain stability in the nomenclature and avoid confusion. Veterinarians may treat the genus name *Prescottella* [48] as a later heterotypic synonym of *Rhodococcus* Zopf 1891 [47] and choose to retain use of *R. equi* over *P. equi*.

## The volume of DNA sequence data and yet-to-be cultured prokaryotes

Metagenomic sequencing approaches have led to a surge in the discovery of new prokaryotes from all manner of sources and environments. The issue of how these new bacteria can be appropriately named and where yet-to-be cultured prokaryotes should be placed in relation to classification was covered by several presenters.

The classification of genomes from yet-to-be cultured and/or ultra-fastidious prokaryotes was addressed by Iain Sutcliffe. It has been estimated that ~85% of prokaryote diversity relates to Archaea and Bacteria that are yet to be cultivated [5]. Under the current rules of the ICNP, a viable pure prokaryote culture has to be available for the valid publication of a name. As a result, fewer than 0.2% of prokaryotes have been named [6]. The SeqCode provides a code of nomenclature based on genome sequence data as the type, with rules adapted from those of the ICNP [7], operated through an online registry (https://seqco.de/). Importantly, the SeqCode complements the ICNP (for example, validly published ICNP names are recognized under the SeqCode) and has the potential to operate in parallel with the ICNP, thereby allowing the formal naming of a vastly expanded range of microbial diversity.

In response to Iain Sutcliffe’s presentation on SeqCode, Markus Göker indicated that SeqCode contravenes the ICNP and that such a proposal had previously been rejected by the ICSP [8]. Göker added that the ICNP has never prevented anyone from naming a prokaryote. Göker was of the opinion that the SeqCode undermines a scientific standard, reduces the incentive to cultivate and deposit strains and that it does not benefit practitioners. An alternative proposal to amend how *Candidatus* names are regulated under the ICNP has since been published, which some consider to obviate the need for the SeqCode [9].

Markus Göker outlined the structure and purpose of the ICSP, its Judicial Commission and the List of Prokaryotic names with Standing in Nomenclature (LPSN) database [10]. He explained that many concepts of the ICNP are misunderstood or may even be misrepresented in the scientific literature [11]. For example, application of the ICNP rules does not usually lead to changes in prokaryotic names. Name changes in databases with respect to prokaryotic nomenclature, however, can be problematic [12] and users are becoming increasingly aware of this discrepancy [13]. Göker further explained his view on taxonomic conservatism: splitting *Borrelia* into *Borrelia* and *Borreliella* [14] was not taxonomically conservative, whereas merging *Ochrobactrum* and *Brucella* was [15]. He proposed a roadmap for mitigating changes in prokaryotic nomenclature that considers these and other issues, such as the possibility of deferring the application of a taxonomically preferable name.

Leighton Pritchard presented genomeRxiv, a genome-based system developed to provide a quantitative, stable and robust organization for prokaryotes, independent of existing nomenclature and taxonomy [16]. Specifically, genomeRxiv maps each unique prokaryote genome to a ‘genome space’ that is subdivided into groups of similar sequences, indexed by unique addresses called LINgroups, based on life identification numbers (LINs), and analogous to map grid references [17]. The rationale of this system is to address (1) differences between microbial taxonomy and nomenclature based on historical polyphasic classifications and quantitative clustering of prokaryotes by genome similarity, (2) the imprecise definitions of some taxonomic ranks (e.g. phylum and class) and (3) limitations of binomial nomenclature, which models the network of prokaryotic evolutionary history as an ever-bifurcating tree. genomeRxiv provides an objective ‘Rosetta Stone’ onto which alternative classifications (e.g. variations in nomenclature, or pathovar information) can be mapped simultaneously, enabling unambiguous translation across multiple schemes. Bona fide species definitions, host range or phenotype can be expressed as collections of one or more LINgroups and compared with classifications made by alternative schemes to establish concordance or difference. An early implementation of this service is publicly available at http://genomerxiv.cs.vt.edu/.

Jake Turnbull and Hannah Macgregor from the National Collection of Type Cultures (NCTC), UK, which serves as a repository for nomenclatural type strains for prokaryotes, presented on the taxonomic composition, accessioning efforts and whole-genome sequencing projects of the collection. They also highlighted the need for a pure culture for valid publication under the ICNP rules. The number of strains accessioned into the NCTC as proposed type strains is increasing each year, as is the amount of whole-genome sequencing data associated with NCTC strains [18] deposited in culture collections; however, culture collections clearly cannot be representative of yet-to-be cultured prokaryote diversity. Interestingly, there is a strong culture collection bias towards pathogens; of the >900 type strains held by the NCTC, 513 are human pathogens. There are notable gaps within the collection for intracellular (*Rickettsia*, *Chlamydia*, *Ehrlichia* and *Coxiella* spp.) and challenging to cultivate (Treponemes, *Leptospira* and *Borrelia* spp.) prokaryotes.

Nicola Holden highlighted some of the potential limitations of a lack of an associated culturable isolate and the use of solely microbiome sequence datasets. Attention needs to be given to the quality and completeness of assembled genomes from microbiomes, so that there are no misunderstandings in how they are reported and the possibility of erroneous designation of a species from an amplicon sequence metabarcode is addressed. A minimum set of requirements for metagenome-assembled genomes (MAGs) is described in [19].

Mark Pallen presented key insights into nomenclature in the age of high-throughput genomics and metagenomics from his recent work addressing bacterial and archaeal taxonomy in this rapidly evolving field. He highlighted the enduring legacy of Linnaeus and Latin binomials, presenting an automated method for generating over a million descriptive names for prokaryotes using combinatorial concatenation of Latin and Greek roots [20]. He then highlighted the slower evolution of prokaryotic nomenclature by comparison, advocating for a system that is more suited to the genomics era, adopting a linguistic pragmatism where naming rules are mandatory but recommendations are optional. Pallen argued that, when coupled with automated naming, this approach could alleviate the need for linguistic expertise among microbiologists and lead to a more inclusive and accessible approach to nomenclature [20]. Most recently, Pallen and collaborators have argued that current approaches to the creation of descriptive prokaryotic names cannot match the dizzying pace of taxonomic discovery and so instead have proposed an innovative solution: the generation of arbitrary but well-formed Latinate names at a scale sufficient to apply names to tens of thousands of newly discovered but as yet unnamed taxa within the Genome Taxonomy Database [21]. In conclusion, Pallen stressed the need for a critical shift towards a more dynamic, automated and inclusive system of microbial nomenclature, aligning with the exponential growth in species discovery in genomic and metagenomic research and the need to engage with a diverse global population of microbiologists.

## Nomenclature change and practical microbiology: the challenges for nomenclature end-users

The potential impacts of nomenclature changes on practical microbiology were presented by end-users of nomenclature changes, with a special focus on clinical microbiology and food safety. Plant pathology and industrial microbiology were not discussed in detail, although some aspects of the discussion may also be relevant to these areas.

The potential negative impact of nomenclature changes on clinical microbiology was exemplified by Susan Butler-Wu in relation to the rapid adoption of new names by diagnostic databases that are used for microbial identification of clinical bacterial isolates. The example of the reclassification of bacteria in the genus *Ochrobactrum* to the genus *Brucella* was highlighted. In contrast to *Ochrobactrum* species, which are opportunistic pathogens with a low-level risk to the wider community, *Brucella* species cause brucellosis, are designated as posing a severe threat to public health and require the implementation of containment and measures to protect laboratory personnel from laboratory-acquired infection. Critically, the antimicrobial treatment of *Brucella* infections differs vastly from that of *Ochrobactrum* species [22].

Conor Meehan presented a proposal for a clear clinical nomenclature of the *Mycobacterium* species associated with human infection. Tuberculosis is primarily caused by members of the *Mycobacterium tuberculosis* complex, which have been renamed, combined and split many times over the decades, and contains both human- and animal-associated lineages, many of which have been designated as separate species in the past. This shifting nomenclature of species within this genus means that different designations have been used clinically, leading to confusion on which terminology should take precedence and which diagnostic test outputs definitely mean that an infection is tuberculosis. The nomenclature system presented proposes ‘tubercle bacilli’ to designate any bacterium causing clinical tuberculosis, with specific species and lineage names only listed when required for diagnostic tests, e.g. those with intrinsic drug resistance. The proposal aims to create a unified clinical nomenclature that would be somewhat agnostic to the shifting bacterial taxonomy underneath, to serve as an example for other infectious diseases with similar issues.

Nicola Holden presented the issues relating to how to describe *Escherichia coli* associated with foodborne disease. In the UK, nearly a million cases of foodborne disease annually are caused by 13 known pathogens, of which Shigatoxigenic *Escherichia coli* (STEC) is a priority pathogen group because the resulting disease can be fatal. *E. coli* comprises a species complex of genetically diverse organisms [23], of which only a small number of subspecies strains can cause disease. Navigation of the different pathogenic *E. coli* types is complicated because different terms describe different contexts. These can relate to the genotype, e.g. STEC; the disease type, e.g. enterohaemorrhagic or enteropathogenic *E. coli* (EHEC, EPEC); or an associated phenotype, e.g. diffuse adhering or adherent-invasive *E. coli* (DAEC, AIEC) [24]. There can be some overlap between the different terms, e.g. some STEC may be described as EHEC, while other STEC do not cause any symptomatic clinical disease.

Genomics of STEC pathogens underpins molecular epidemiology and disease outbreak tracing [25]. For disease outbreak investigation, the ‘SNP’ address system is recognized as the gold standard [26]. The emergence of genetically diverse non-O157 STEC serotypes, many of which cannot cause disease, has led to the need for a genome-based description of their virulence factors and assignment of risk potential e.g. Scottish *E. coli* O157/STEC Reference Laboratory (SERL) Report 2020 https://www.foodstandards.gov.scot/publications-and-research/publications/whole-genome-sequence-typing-and-analysis-of-non-o157-stec). Furthermore, detection of *E. coli* as a hygiene indicator in environmental matrices or water is taken to indicate animal (normally human) faecal contamination, despite the body of evidence showing they are a normal part of many environmental communities [27,28]. Therefore, there is an absolute requirement to use correct and helpful descriptions of members of the species. The application of genome-based investigation for food safety and quality has yet to be widely adopted as it has been for clinical disease, although the promise exists.

Angelika Kiepas highlighted the importance of robust nomenclature in relation to *Streptomyces*, a pharmaceutically important genus and a potential source of novel antibiotics [29,29]. Classifying *Streptomyces* into ‘genus’ or ‘species’ clusters using a single 16S rRNA results in poor phylogenetic resolution and inappropriate grouping of genomes into clusters of variable relatedness, with inclusion of distantly-related organisms [30]. Efficient drug discovery, avoiding rediscovery, employs comparative genomics and pangenomic studies to discover novel antibiotics produced by closely related organisms [31]. Accurate, consistent clustering for pangenomics is most reliably achieved with the use of whole-genome sequence difference estimates. These resolve *Streptomyces* into potentially six novel genera [1]. Genome-based comparative genomic analyses are least likely to bias pangenomic analyses or misdirect efforts to discover novel antimicrobial capabilities.

Sybren de Hoog outlined the relatively recent global consensus framework for mitigating the impact of nomenclature changes of medically important fungi [32]. He highlighted the impacts of both genus and species name changes and described the implementation of a standing committee to routinely review name changes of medically important fungi as well as an open access database with current and prior names. Critically, this framework also stipulates that a name should be recommended for end-users by a committee under the auspices of the International Society for Human and Animal Mycology, Working Groups on Nomenclature and Fungal Diagnostics (www.atlasclinicalfungi.org). The multi-disciplinary work of de Hoog and colleagues represents a possible model that could be emulated to deal with the impact of nomenclature changes in bacteria of medical importance.

## Summary of meeting participants’ discussion

The attendees assessed whether the process to designate ‘validly published’ status should be made more meaningful and understandable to both taxonomists and non-taxonomists. One important outcome of the meeting was a broad recognition that there is substantial confusion and misunderstanding of what a valid publication represents. The process of assigning the status ‘validly published’ to a name provides a scientific standard or a ‘quality stamp’ to the name, though not necessarily to the taxonomic opinion leading to the proposal of a name. It also ensures the collection of information to accompany the name, including a protologue (formal description of the name, origin of the name, properties of the taxon and more) and being associated with a designated type strain, which must be culturable and deposited in two culture collections to enable broad scientific access (Box 2). The requirements for valid publication of a name are delineated in the ICNP [33], which provides a framework for how to arrive at a name, but does not prescribe taxonomic methods and does support taxonomic freedom. Today, being validly published is often misinterpreted by many non-taxonomists and some taxonomists to mean the ‘name that should be used’ when actually we should be interpreting a validly published name for a taxon to mean a ‘name that may be used’. Thus, when considering competing validly published names for the same taxon, the ‘correct name’ to be chosen is a matter of taxonomic opinion (Box 3). Under the ICNP, illegitimate names (those contrary to a Rule or Rules) and names rejected by the Judicial Commission must not be used. Other names may have been proposed and ‘effectively published’ [34], but these lack formal standing in nomenclature and validation of such names is strongly encouraged.

Whether or not a microbe can be defined solely by its genome sequence fuelled animated debate; while the power of whole-genome phylogenetics is undisputed in its application to taxonomy, NCTC delegates hold the view that microbes are more than computational representations of their genomes and that the use of type cultures in prokaryotic taxonomy is of high value. This latter point is not disputed by proponents of the SeqCode, who nevertheless believe that a genome sequence can be sufficient in itself to serve as type [7,35].

Regarding the discussion topic of using computational approaches to generate names for microbes, a clear advantage is that a high enough volume of distinct ICNP-compliant names could be produced to be applied to the hypothesized number of microbial species. However, there is some understandable concern regarding a huge proliferation of names creating confusion and that computationally generated nomenclature may diminish names of intrinsic meaning, although it is noted that the ICNP permits arbitrary names and Principle 4 states that the ‘purpose of giving a name to a taxon is to supply a means of referring to it rather than to indicate the characters or the history of the taxon’.

The NCTC recognizes its place in proliferating a better understanding of prokaryote nomenclature and is supportive of efforts to prevent confusion consequent to name changes to microbiologists.

Concern was raised about the highly variable quality of genomes, both new assemblies and many that have been deposited in well-known databases and the impact of this on genome-based taxonomy. Therefore, it was suggested that ‘genomic standards for description of novel species’ should be updated by IJSEM to reflect current technologies. This short report would serve as a guide to authors on how to generate their data to the highest standard (e.g. using a combination of long and short-read sequencing chemistries) and how to computationally analyse and assemble the results. Considering the latter of these issues, it was recommended that submissions should include quality control statistics such as BUSCO or CheckM [36,37], information about detection of plasmids [38], read depth and sequence data from ribosomal genes. It was noted that not all researchers benefit from bioinformatic support and that any such proposal should account for this by indicating software tools that are publicly available via a web browser or using platforms such as Galaxy [39]. It is noted that, subsequent to the meeting, useful update guidelines have been published [40]. Another issue for taxonomists and non-taxonomists is the lack of consensus around the delineation of genera based on whole-genome sequencing.

Reliance on molecular data and predictive genome annotation methods also highlighted a concern that the microbiology community is alienating itself from its heritage knowledge base. Prior to the advent of ubiquitous high-throughput sequencing, bacteria were described and identified by a suite of biochemical and phenotypic tests. Historically, these data have been collated in Bergey’s Manual of Systematic Bacteriology of Archaea and Bacteria. The wealth of phenotypic and descriptive detail in the manual is exemplified by the timescale for publication of the five volumes (>5,000 pages) of the second edition which took from 2001 to 2012 to complete. The online [41] version is continually updated and remains the key reference source for prokaryote description and taxonomy. It was felt that computational innovations may be able to afford interrogation of these historical data with a draft genome search query. Where a MAG is used as a search query, this could improve the chances of axenic culture by providing a prediction of growth requirements such as carbon and nitrogen source utilization.

There was general agreement that, without formal regulation such as that provided by the ICNP, the landscape of competing classifications has the potential to lead to further taxonomic confusion [42].

The role of genome sequencing in the future of prokaryote taxonomy was a particular point of contention and highlighted the chasm between and among taxonomists, nomenclature experts and practical/applied microbiologists. It can be argued that because species have been defined for decades or more prior to the advent of genome sequencing, genome sequences are not essential for species definition. Conversely, the argument that relatively inexpensive and reliable sequencing methodologies exist today and *should* be used to define species can be made. Just because something has ‘always been done’ a certain way does not make it right. Regardless of our areas of study, we are all scientists. Advances are not made simply by doing things the way they have always been done.

In relation to practical microbiology, the potentially negative consequences for patients when a new, validly published name is reported clinically but is unrecognized by the clinician caring for the patient need to be acknowledged. From a clinical perspective, the argument that a clinician not aware of a new name ‘should be educated’ was considered to reflect a lack of understanding of the professional situation of most clinicians.

Rapid improvements in sequencing technology have allowed for the more precise genomic definition of species. While 16S rRNA gene sequencing has been incredibly useful with regard to bacterial classification, both the inability to reliably differentiate organisms to the species level and its low discriminatory power for many genera are well-documented [43,44]. While re-classifying prokaryotes based on more uniform standards that include higher-powered metagenomic sequencing technology may seem like the most logical and perhaps scientifically sound approach, from a clinical microbiology perspective, it could potentially negatively impact patient care where a genus or species name is unfamiliar to the clinician or where a particular name carries with it particular antimicrobial treatment or clinical management differences.

## Conclusions

The bringing together of key stakeholders, taxonomists, clinical microbiologists, biological resource centre colleagues and industry, to enable group discussion and reporting thereof, was the crucial starting point to approach a solution. Industry manufacturers of *in vitro* diagnostics tests and devices, in particular, must also be active participants in these conversations as their databases are a critical tool in many clinical laboratories [45]. There was a consensus that we must work together to arrive at a compromise that will ultimately put patient health and safety at the forefront, but that will do so in a manner that maintains taxonomic freedom.

There was agreement that the medical mycology model of a global committee providing a recommended name for end-users, particularly in relation to infectious diseases/clinical microbiology, could be applied to prokaryotes, with the potential to be included in the LPSN database [10].

## Meeting outcome

The Ad Hoc Committee for Mitigating Changes in Prokaryotic Nomenclature was initiated in January 2024 under the auspices of the ICSP to develop a framework leading to a recommended name for end-users of prokaryote nomenclature [46] (https://www.the-icsp.org/index.php/ad-hoc-committee-on-mitigating-name-changes-of-prokaryotes).

## Abbreviations

ICNP: International Code of Nomenclature of Prokaryotes
ICSP: International Committee on Systematics for Prokaryotes
LINs: life identification numbers
LPSN: List of Prokaryotic names with Standing in Nomenclature
MAGs: metagenome-assembled genomes
NCTC: National Collection of Type Cultures
STEC: Shigatoxigenic *Escherichia coli*
